# Luminescence Enhancement and Temperature Sensing Properties of Hybrid Bismuth Halides Achieved via Tuning Organic Cations

**DOI:** 10.3390/molecules28052380

**Published:** 2023-03-04

**Authors:** Ting-Hui Zhuang, Yi-Min Lin, Hao-Wei Lin, Yan-Ling Guo, Zi-Wei Li, Ke-Zhao Du, Ze-Ping Wang, Xiao-Ying Huang

**Affiliations:** 1 Fujian Provincial Key Laboratory of Advanced Materials Oriented Chemical Engineering, College of Chemistry and Materials Science, Fujian Normal University, Fuzhou 350007, China; 2 State Key Laboratory of Structural Chemistry, Fujian Institute of Research on the Structure of Matter, Chinese Academy of Sciences, Fuzhou 350002, China; 3 College of Chemistry, Fuzhou University, Fuzhou 350116, China

**Keywords:** bismuth, inorganic-organic, luminescence, ionic liquid, temperature sensing

## Abstract

Bismuth-halide-based inorganic-organic hybrid materials (Bi-IOHMs) are desirable in luminescence-related applications due to their advantages such as low toxicity and chemical stability. Herein, two Bi-IOHMs of [Bpy][BiCl_4_(Phen)] (**1**, Bpy = *N*-butylpyridinium, Phen = 1,10-phenanthroline) and [PP14][BiCl_4_(Phen)]·0.25H_2_O (**2**, PP14 = *N*-butyl-*N*-methylpiperidinium), containing different ionic liquid cations and same anionic units, have been synthesized and characterized. Single-crystal X-ray diffraction reveals that compounds **1** and **2** crystallize in the monoclinic space group of *P*2_1_/*c* and *P*2_1_, respectively. They both possess zero-dimensional ionic structures and exhibit phosphorescence at room temperature upon excitation of UV light (375 nm for **1**, 390 nm for **2**), with microsecond lifetime (24.13 μs for **1** and 95.37 μs for **2**). Hirshfeld surface analysis has been utilized to visually exhibit the different packing motifs and intermolecular interactions in **1** and **2**. The variation in ionic liquids makes compound **2** have a more rigid supramolecular structure than **1**, resulting in a significant enhancement in photoluminescence quantum yield (PLQY), that is, 0.68% for **1** and 33.24% for **2**. In addition, the ratio of the emission intensities for compounds **1** and **2** shows a correlation with temperature. This work provides new insight into luminescence enhancement and temperature sensing applications involving Bi-IOHMs.

## 1. Introduction

In recent years, inorganic-organic hybrid materials (IOHMs) have received much attention due to their designable dual functionalities of inorganic and organic moieties [1,2,3]. IOHMs are widely developed for applications in ferroelectricity [4,5], photovoltaics [6], proton conduction [7], solar cells [8], photocatalysts [9], scintillations [2,10], and luminescence [3,11,12,13,14]. Zero-dimensional (0D) metal halide-based IOHMs have diverse functionalities for photoluminescent applications due to the diversity of organic cations and the geometric configurations of anionic halometalate units [13,15]. Therefore, the desired luminescent properties can be realized via the modulation of cationic or anionic moieties [2,15]. For the anionic units, the choice of halometalate unit greatly affects the luminescent properties of the compound [16]. For example, the Xia group reports on three IOHMs with the same cation and different anionic units, namely (EnrofloH_2_)Pb_2_Cl_6_·H_2_O, (EnrofloH_2_)BiCl_5_·Cl·2(H_2_O)·H_3_O, and (EnrofloH_2_)SnCl_6_·H_2_O (Enroflo = enrofloxacin), that exhibit different photoluminescence [16]. Organic cationic moieties, on the other hand, can influence the supramolecular structure of IOHMs and the stacking modes of anionic units, thus altering the photoluminescent properties of IOHMs [2,10,17]. For instance, in 2019 our group reported that the variance of cations in manganese-based IOHMs led to the difference in photoluminescence quantum yield (PLQY) for [P14]_2_[MnBr_4_] (PLQY = 81%, P14 = *N*-butyl-*N*-methylpyrrolidinium) and [PP14]_2_[MnBr_4_] (PLQY = 55%, PP14 = *N*-butyl-*N*-methylpiperidinium) [18]. In 2021, our group reported two bismuth halide-based IOHMs (Bi-IOHMs) with distinct organic cations, namely [Emim]BiCl_4_(bp2do) and [Emmim]BiCl_4_(bp2do) (Emim = 1-ethyl-3-methylimidazolium, Emmim = 1-ethyl-2,3-dimethylimidazolium, bp2do = 2,2′-bipyridyl-1,1′-dioxide) [2]. It was found that the change in the number of substituents in the organic cations accounted for the change of their PLQY, i.e., 2.31% for [Emmim]BiCl_4_(bp2do), and 25.05% for [Emim]BiCl_4_(bp2do).

Ionic liquids are “green” solvents with characteristics of non-volatility, low melting point, high thermal stability, and designability [19]. Traditionally, ionic liquid cations (ILCs) are nonsymmetrical organic species, of which the cation core has appended to one or more organic groups [20]. By combining luminescent metal halide anionic units with ILCs, novel luminescent materials based on ILCs can be obtained. Notably, luminescent 0D IOHMs with ILCs have abundant supramolecular interactions, such as hydrogen bonding, anion···π interactions, and π···π interactions, which favors the tuning of their photoluminescence [12,18,21].

Among various luminescent 0D IOMHs, the Bi-IOHMs are extremely desirable due to their low toxicity and chemical stability [22,23]. Meanwhile, the introduction of heavy atomic Bi atoms can enhance the spin-orbit coupling and, thus, improve the intersystem crossing, which facilitates phosphorescence at room temperature [24]. Furthermore, the Bi(III) ion is capable of combining N and O atoms of organic ligands, resulting in the organically modified halobismuthates and the intensification of luminescence [2,10,11,13,25].

**Scheme 1:**
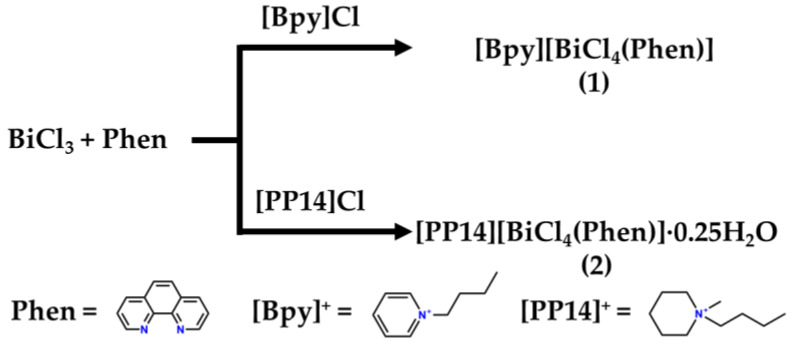
The synthetic routes for title compounds.

Motivated by the current situation mentioned above, two Bi-IOHMs, namely [Bpy][BiCl_4_(Phen)] (**1**, Bpy = *N*-butylpyridinium, Phen = 1,10-phenanthroline) and [PP14][BiCl_4_(Phen)]·0.25H_2_O (**2**), have been synthesized (Scheme 1). The title compounds contain the same anion of the Phen-coordinating halobismuthate unit and distinct ILCs. Their crystal structures and photophysical properties have been studied and compared carefully. The variation in ILCs causes discrepancies in supramolecular interactions in title compounds, resulting in different rigidity of the structure. As a result, the title compounds show distinct PLQY (0.68% for **1,** and 33.24% for **2**). Meanwhile, it is interesting to note that both compounds **1** and **2** exhibit temperature-sensing properties. The ratio of the intensity of the high-energy peak to the low-energy peak versus temperature for **1** and **2** can be well fitted by the liner function and exponential function, respectively. This work enhances the photoluminescence of Bi-IOHMs by modulating the cations and discovering their potential as temperature-sensing materials. To our knowledge, this is the first report on the temperature-sensing performance of Bi-IOHMs, providing a new option for the development of temperature-sensing materials [26].

## 2. Results and Discussion

### 2.1. Crystal Structure Descriptions

Single crystals of title compounds were obtained via the solvothermal method using acetonitrile as the solvent (Scheme 1). Single crystal data of title compounds were recorded at 100 K. The crystallographic data and structural refinement details, selected bond lengths and bond angles for **1** and **2**, as well as hydrogen bonding data for **1** and **2** are listed in Tables S1–S5, respectively. The π···π, anion···π, and C–H···π interactions in structures **1** and **2** at 100 K are depicted in Tables S6–S8. Crystal Data for [Bpy][BiCl_4_(Phen)] (**1,** C_21_H_22_BiCl_4_N_3_, *M* = 667.19 g/mol): monoclinic, space group *P*2_1_/*c* (no. 14), *a* = 10.1667(7) Å, *b* = 15.5703(7) Å, *c* = 15.1177(8) Å, *β* = 105.882(7)°, *V* = 2301.8(2) Å^3^, *Z* = 4, *T* = 100(2) K, *μ*(Mo*Kα*) = 8.137 mm^−1^, *D*_calc_ = 1.925 g/cm^3^, 26,752 reflections measured (5.506° ≤ 2*θ* ≤ 59.214°), 5751 unique (*R*_int_ = 0.0845, *R*_sigma_ = 0.0663) which were used in all calculations. The final *R*_1_ was 0.0361 (*I* > 2σ(*I*)) and *wR*_2_ was 0.0777 (all data). Crystal Data for [PP14][BiCl_4_(Phen)]·0.25H_2_O (**2**, C_22_H_30.50_BiC_l4_N_3_O_0.25_, *M* = 691.77 g/mol): monoclinic, space group *P*2_1_ (no. 4), *a* = 11.3962(9) Å, *b* = 18.0531(15) Å, *c* = 12.2968(8) Å, *β* = 91.253(7)°, *V* = 2529.3(3) Å^3^, *Z* = 4, *T* = 100(2) K, *μ*(Mo*Kα*) = 8.137 mm^−1^, *D*_calc_ = 1.817 g/cm^3^, 25,656 reflections measured (4.008° ≤ 2*θ* ≤ 50.040°), 8866 unique (*R*_int_ = 0.0763, *R*_sigma_ = 0.0893), which were used in all calculations. The final *R*_1_ was 0.0416 (*I* > 2σ(*I*)) and *wR*_2_ was 0.0771 (all data).

**Figure 1:**
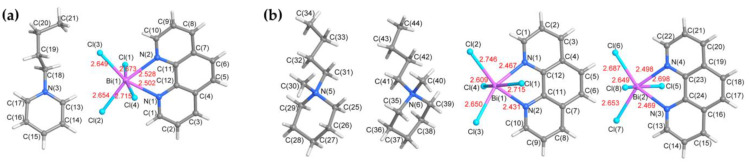
(**a**) The [Bpy]^+^ cation and [BiCl_4_(Phen)]^−^ anion of **1**. (**b**) The [PP14]^+^ cations and [BiCl_4_(Phen)]^−^ anions of **2**.

The asymmetric unit of **1** consists of one bismuth atom, four chloride atoms, one Phen molecule, and one ILC unit [Bpy]^+^ exhibiting statistical distribution (Figure S1). As shown in Figure 1a, the Bi atom is six-coordinated connecting to four Cl atoms as well as two N atoms from the ligand Phen in a chelating mode. The Bi-Cl bond lengths range from 2.6488(12) to 2.7148(15) Å, while the Bi-N bond lengths are 2.501(4) and 2.528(3) Å (Table S2). The Bi-Cl and Bi-N bond lengths are comparable with those in the previously reported bismuth chlorides [11,27,28]. The asymmetric unit of compound **2** consists of two [BiCl_4_(Phen)]^−^ anionic units, two ILCs [PP14]^+^, as well as half an H_2_O molecule (Figure S2). The coordination geometry for the [BiCl_4_(Phen)]^−^ units are almost identical to each other and also is the same as that in compound **1** (Figure 1b, Table S3). The Bi-Cl bond lengths range from 2.609(4) to 2.746(4) Å. Meanwhile, the Bi-N bond lengths range from 2.431 (12) to 2.498(12) Å, which is slightly shorter than previously reported [11,27,28].

**Figure 2:**
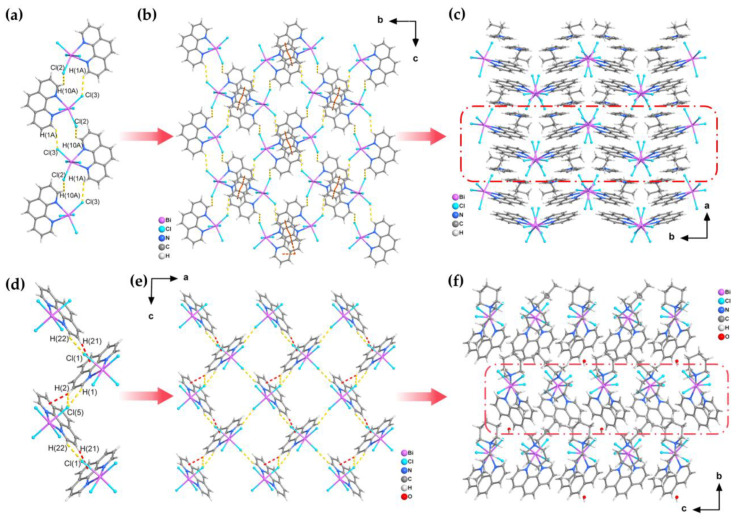
(**a**) A diagram showing that the anionic units in **1** are connected to each other to form a 1D chain by hydrogen bonds (yellow dotted lines); (**b**) a diagram showing the 2D layer in **1** formed by inter-chain π···π interactions (orange dotted lines) viewed along the *a* axis; (**c**) packing diagram of **1** viewed along the *c* axis; (**d**) A diagram showing that the anionic units in **2** are connected by hydrogen bonds (yellow dotted lines) and C-H···π interactions (red dotted lines) to form a 1D chain; (**e**) A diagram showing the 2D layer in **2** formed by inter-chain hydrogen bonds (yellow dotted lines) viewed along the *b* axis; (**f**) packing diagram of **2** viewed along the *a* axis.

The two compounds display different supramolecular packing modes (Figure 2). Both compounds **1** and **2** exhibit a 3D supramolecular framework by supramolecular interactions. The supramolecular interactions in compounds **1** and **2** have been contrasted carefully. **1** and **2** show abundant hydrogen bonds, however, the **2** possess stronger hydrogen bonds (ranging from 2.76 to 2.98 Å, Table S5) among the anionic units than **1** (ranging from 2.88 to 2.99 Å, Table S4). **1** possesses many π···π interactions with the distance range from 3.604(14) to 3.963(14) Å (Table S6), while no π···π interaction exists in **2**. Compound **1** possesses the anion···π interaction between Cl(3) atom to pyridine rings from the [Bpy]^+^ (Cl(3)···N(3A)→C(13A)→C(14A)→C(15A)→C(16A)→C(17A), Cl(3)···N(3B)→C(13B)→C(14B)→C(15B)→C(16B)→C(17B)). Meanwhile, compound **2** has four unique C-H···π interactions between C-H groups from phen rings or cations and π-conjugated rings of Phen (Table S8, C(2)-H(2)···N(3)→C(13)→C(14)→C(15)→C(16)→C(24), C(21)-H(21)···C(4)→C(5)→C(6)→C(7)→C(11)→C(12), C(26)-H(26A)···C(4)→C(5)→C(6)→C(7)→C(11)→C(12), and C(44)-H(44A)···N(3)→C(13)→C(14)→C(15)→C(16)→C(24)).

For compound **1**, the anionic [BiCl_4_(Phen)]^−^ units are connected by hydrogen bonds (C(1)-H(1A)···Cl(3)#1 and C(10)-H(10A)···Cl(2)#2) to form one-dimensional (1D) chains (Figure 2a, Figure S3 and Table S4, symmetry codes: #1 x, −y + 1 / 2, z + 1 / 2; #2 x, −y + 1 / 2, z − 1 / 2). The two-dimensional (2D) anionic layer could be formed by π···π interaction linking these 1D chains (Figure 2b, Figure S4, and Table S6). Ultimately, the 2D anionic layer forms a 3D supramolecular framework via hydrogen bonds, π···π interaction, as well as anion···π between the anionic and cationic units (Figure 2c, Figures S5–S7, Tables S4, S6, and S7).

For compound **2**, the anionic [BiCl_4_(Phen)]^−^ units are connected by hydrogen bonds and C-H···π interactions, namely C(1)-H(1)···Cl(5)#1, C(2)-H(2)···Cl(5)#1, C(22)-H(22)···Cl(1)#4, C(2)-H(2)···Cg6, and C(21)-H(21)···Cg4 (Tables S5 and S8, Figure S8 and Figure 2d, symmetry codes: #1 −x + 1, y + 1 / 2, −z, #4 −x + 1, y − 1 / 2, −z + 1; Cg(4): C(4)→C(5)→C(6)→C(7)→C(11)→C(12); Cg(6): N(3)→C(13)→C(14)→C(15)→C(16)→C(24)), to form one-dimensional (1D) chains. The 2D anionic layer could be formed by hydrogen bonds (C(10)-H(10)···Cl(8)#2, C(13)-H(13)···Cl(4)#3) among anionic units linking these 1D chains (Figure 2e, symmetry codes: #2 −x, y + 1 / 2, −z + 1; #3 −x, y − 1 / 2, −z). Furthermore, the anion layers are connected to the cationic [PP14]^+^ units by abundant hydrogen bonds (Figure S9 and Table S5). Finally, a 3D supramolecular framework is formed by connecting the adjacent 2D layers via C-H···π interactions between anionic and cationic units (Figure 2f, Figure S10, and Table S5).

### 2.2. Hirshfeld Surface Analyses

**Figure 3:**
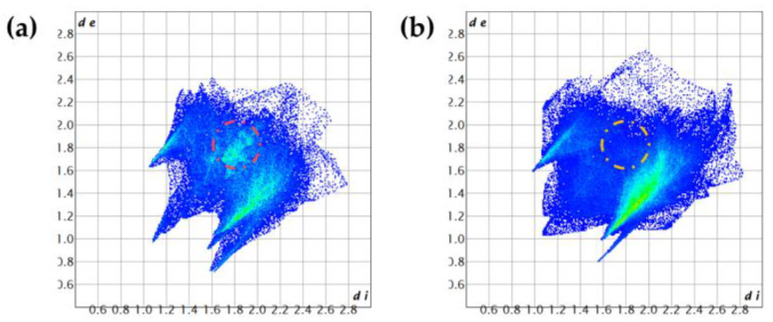
2D finger plots for the [BiCl_4_(Phen)]^−^ anion in compounds **1** (**a**) and **2** (**b**).

Hirshfeld surface analyses and 2D fingerprint plots have been utilized to visually exhibit the different packing motifs and intermolecular interactions in **1** and **2** (Figure 3). The red, yellow, green, as well as blue colors indicate the relatively strong to relatively weak interactions sequentially. According to the comparative 2D fingerprint plots of [BiCl_4_(Phen)]^−^ anion in compounds **1** and **2**, it is clear that a more ample supramolecular interaction exists in compound **2** relative to **1**, which suggests that compound **2** has a more rigid structure than **1**, despite the π···π interaction (circled by red/yellow dashed lines) existing only in **1**.

### 2.3. PXRD and Thermal Stability Analyses

The experimental powder X-ray diffraction (PXRD) patterns for compounds **1** and **2** match well with the corresponding simulated ones, confirming the high purity of each obtained phase (Figures S11 and S12). Thermogravimetric (TG) analysis was performed to investigate the thermal stability of compounds **1** and **2** (Figures S13 and S14). Compound **1** underwent two continuous steps of weight loss in the range of 240–580 °C, which could be attributed to the loss of ILCs, organic ligand Phen, and partial bismuth chlorides [2]. Compound **2** mainly underwent one step of weight loss in the range of 250–450 °C, while a slight weight loss of nearly 0.9% due to the escape of the lattice H_2_O molecules was observed at around 100–150 °C.

### 2.4. Optical Property

Solid-state optical absorption spectra of compounds **1** and **2** were performed and are listed in Figures S15 and S16, respectively. The absorption edges for **1** and **2** are 2.83 and 2.96 eV, respectively. These absorptions mainly are attributed to the inorganic-organic charge-transfer (IOCT), as previously reported for Bi-IOHMs [2,10]

### 2.5. Photoluminescence

**Figure 4:**
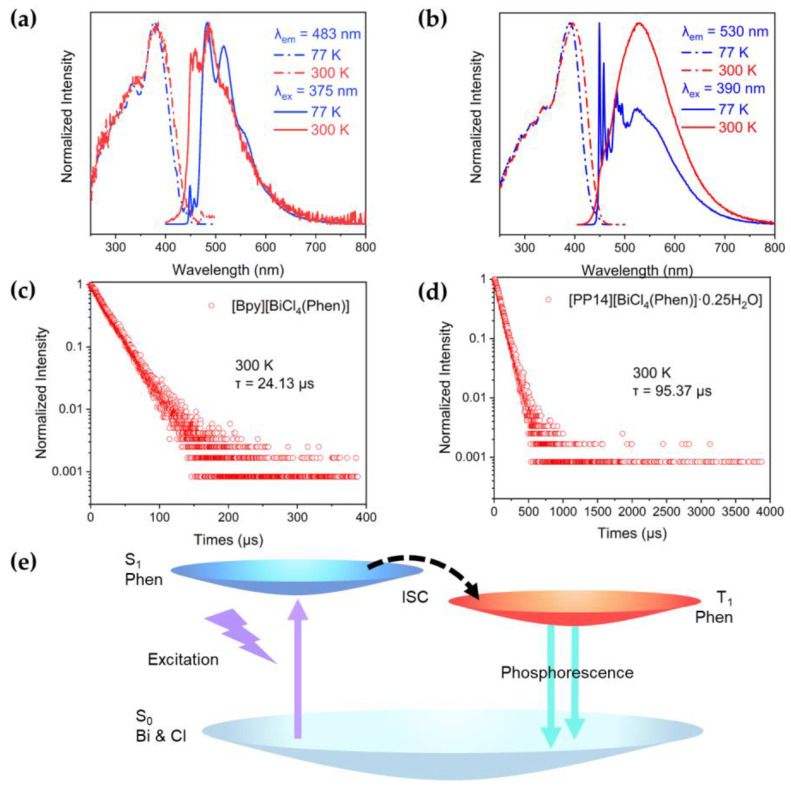
The PLE and PL spectra for **1** (**a**) and 2 (**b**); The PL decay spectra at 300 K for **1** (**c**) and **2** (**d**); The diagram of the photoluminescence mechanism (**e**).

The photoluminescence (PL) spectra, photoluminescence excitation (PLE) spectra, and time-resolved PL spectra have been performed to investigate the photoluminescent properties of compounds **1** and **2** (Figure 4). Under the excitation of UV light, compound **1** shows weak cyan emission, while compound **2** exhibits bright cyan emission (Figures S17 and S18). Both compounds **1** and **2** possess wide excitation peaks from the UV light region to the blue light region (250 to 450 nm). At room temperature, the emission for **1** ranges from 430 nm to 700 nm with the maximum peak at 483 nm under the excitation of 375 nm light. The emission for **2** ranges from 420 nm to 800 nm with the maximum peak at 530 nm under the excitation of 390 nm light. The emission peaks of compounds **1** and **2** display gradually clear and sharper vibrational structures as the temperature decreases. These phenomena are consistent with previous reports for Bi-based complexes [29,30], which resulted from the increased ligand rigidity with metal coordination [31]. It is noteworthy that compound **2** has a significantly clearer and sharper vibrational structure than that of **1** at 77 K, which could be attributed to the fact that compound **2** possesses a more rigid structure.

Moreover, PLQY was measured to be 0.68% for **1** and 33.24% for **2** (Figures S19 and S20). The significant distinction between PLQYs of compounds **1** and **2** could be caused by three reasons as previous reports on Bi-IOHMs: (i) the more rigid supramolecular structure of **2** facilitates the suppression of non-radiative transitions; (ii) the slightly longer Bi–N bond lengths for **1** (Bi–N: 2.501(4) and 2.528(3) Å) than **2** (Bi–N: 2.431(12), 2.467(12), 2.469(12), and 2.498(12) Å) could lead to the increasing chance of exciton quenching owing to the long pathway for charge transfer [2]; (iii) the strong π···π interaction for **1** probable lead to the promotion of exciton quenching as generally thought [32].

The PL lifetime (τ) of **1** and **2** was evaluated using time-resolved PL spectra at 300 K. As shown in Figure 4c,d, both the PL decay curves of the two compounds are best fitted to first-order exponential decay function with PL lifetime constants of 24.13 μs and 95.37 μs for **1** and **2**, respectively. Thus, the emissions of **1** and **2** could be attributed to phosphorescence from the triplet energy level of organic ligand Phen.

Further, the probable mechanism of the PL process is summarized as illustrated in Figure 4e: (i) under UV light excitation, the electrons in the ground state of the inorganic moiety will be excited to the singlet state of the organic ligand Phen; (ii) then the electrons will transfer to the triplet excited state of Phen via the intersystem crossing (ISC) process, which is enhanced by the heavy atom effect of Bi atoms; (iii) eventually, the electrons at the triplet excited-state fall back to the ground-state, leading to phosphorescent emission.

### 2.6. Potential for Application to Temperature Sensing

Temperature is one of the most fundamental thermodynamic state parameters, which is of great importance either in industrial production or scientific research. Luminous materials are the main choice of materials for non-contact thermometers due to high detection sensitivity, non-invasiveness, fast response time, and stability [33]. Especially, luminescent materials with a multi-emission are considered a desirable candidate due to their extraordinary accuracy and facility for temperature sensing [34]. Measuring temperature by the ratio of different emission peaks or integrated intensities has the advantage of less dependence on the measurement conditions, and less measurement error from other parameters (such as excitation source power fluctuations, fluorescence detection loss, and atmospheric pressure changes) [33]. Compounds **1** and **2** have well-defined vibrational satellites at low temperatures, making them potentially applicable for temperature sensing.

**Figure 5:**
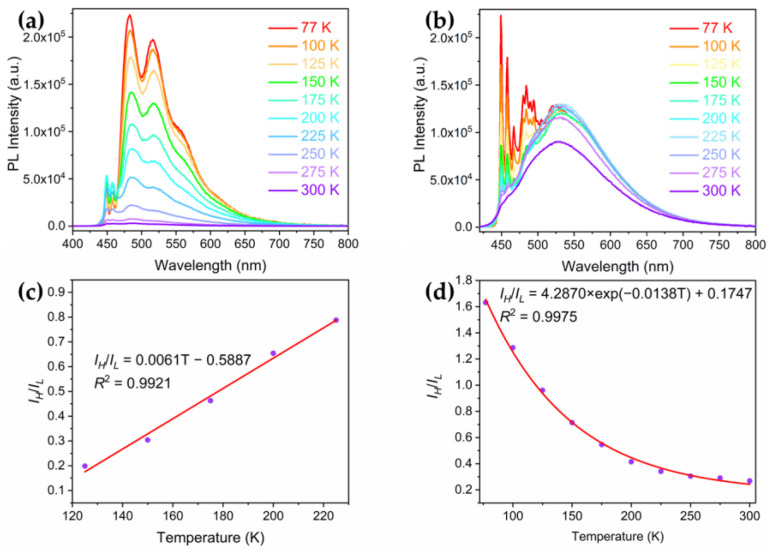
The PL spectra for **1** (**a**) and 2 (**b**). The working curves and equations of *I_H_/I_L_* to temperature for **1** (**c**) and **2** (**d**).

To evaluate the possible application of compounds **1** and **2** as a low-temperature thermometer, further temperature-dependent emission spectra have been measured between 300 and 77 K (Figure 5a,b). For compound **1**, the intensity of the high-energy emission peak near 450 nm (*I_H_*) first increases with increasing temperature and reaches a maximum at 200 K, and then starts to decrease; the intensity of the low-energy emission peak (*I_L_*) near 483 nm, however, keeps decreasing with increasing temperature in the range of 77 K to 300 K. As a result, the ratio of *I_H_* and *I_L_* (*I_H_*/*I_L_*) gradually increased with increasing temperature in the range of 77 K to 300 K. For compound **2**, the intensity of high-energy emission peak near 450 nm and low-energy emission peak near 533 nm also show different trends with the temperature increasing. As the temperature increases from 77 to 300 K, *I_H_* gradually decreases, while *I_L_* barely changes in the range of 77–175 K, then starts to increase and reaches a maximum at 225 K, and finally starts to decrease. Therefore, the *I_H_*/*I_L_* gradually decrease with the temperature increasing from 77 to 300 K. In addition, the relationship between *I_H_*/*I_L_* and temperature has been calculated. For compound **1**, the *I_H_*/*I_L_* values are fitted to the liner function of *I_H_*/*I_L_* = 0.0061*T*–0.5887 (*R*^2^ = 0.9921) in the temperature range from 100 to 225 K (Figure 5c). For compound **2**, the value is best fitted to the exponential function of *I_H_*/*I_L_* = 4.2870 * exp(−0.0138*T*) + 0.1747 (*R*^2^ = 0.9975) in the temperature range of 77–300 K (Figure 5d). Thus, compounds **1** and **2** are both able to respond to temperature as thermometers by measuring the ratio of *I_H_* and *I_L_*. To our knowledge, this is the first temperature-sensing investigation on Bi-IOHMs.

## 3. Conclusions

In summary, two Bi-IOHMs with different ILCs and the same anionic unit have been synthesized. They show different packing modes and supramolecular structures. The choice of ILCs greatly affects the luminescence properties of the compounds. Both compounds **1** and **2** exhibit phosphorescence with vibrational structures, however, compound **2** has about 49-folds of PLQY (33.24%) relative to compound **1** (0.68%). Furthermore, both compounds **1** and **2** exhibit temperature-sensing properties. The ratio between the intensities of the high- and low-energy emissions has a digital relationship with temperature. This work provides new ideas for achieving non-toxic, environmentally friendly, chemically stable, and inexpensive temperature-sensing luminescent materials.

## 4. Materials and Methods

All purchased reagents were utilized directly without further purification. The detailed information for the reagents is listed as follows: bismuth(III) chloride (BiCl_3_, 99%, Vetec, Sigma-Aldrich, St. Louis, MO, USA); 1,10-phenanthroline monohydrate (Phen·H_2_O, AR, Aladdin, Bay City, MI, USA); *N*-butyl-*N*-methyl-piperidinium chloride ([PP14]Cl, 99%+, Lanzhou Greenchem ILs, Lanzhou, China); 1-butylpyridinium chloride ([Bpy]Cl, 99%, Lanzhou Greenchem ILs, Lanzhou, China); acetonitrile (MeCN, 99%, Sinopharm Chemical Reagent Co., Ltd., Shanghai, China).

**Synthesis of [Bpy][BiCl_4_(Phen)] (1).** A mixture of [Bpy]Cl (0.2 mmol), Phen·H_2_O (0.2 mmol), BiCl_3_ (0.2 mmol), and 4 mL acetonitrile were added to a 20 mL Teflon-lined stainless steel autoclave. The container was sealed and heated at 100 °C for 4 days. After naturally cooling to room temperature (RT), yellowish-green block-like crystals were obtained. Yield: ca. 79.0% (based on the Bi atom).

**Synthesis of [PP14][BiCl_4_(Phen)]·0.25H_2_O (2).** The synthetic method is similar to that for **1**, except that the [Bpy]Cl was replaced by [PP14]Cl, and the volume of acetonitrile was increased from 4 mL to 5 mL. After naturally cooling to RT, nearly colorless plate-like crystals were obtained. Yield: ca. 28.9% (based on Bi atom).

**X-ray crystallography**. A single crystal suitable for single-crystal X-ray diffraction (SCXRD) was selected under an optical microscope. Single-crystal data of **1** and **2** were measured and collected on a Supernova CCD diffractometer with graphite-monochromated Mo*K*α radiation (*λ* = 0.71073 Å) at 100 K. The structure was solved by direct methods and refined with full-matrix least squares on *F^2^* by the *SHELX2018* package [35]. π···π, anion···π, and C–H···π interactions were calculated via Platon [36]. CCDC NO. 2,239,428 (for **1**) and 2,239,429 (for **2**) contain the supplementary crystallographic data for this paper. These data can be obtained free of charge from The Cambridge Crystallographic Data Centre via www.ccdc.cam.ac.uk/data_request/cif (accessed on 2 February 2023).

**Characterization.** Powder X-ray diffraction (PXRD) patterns were performed on a Rigaku Miniflex-II diffractometer with Cu *Kα* radiation (*λ* = 1.54178 Å) in the angular range of 2*θ* = 5–65°. The simulated PXRD pattern is calculated from the SCXRD data using the *Mercury* program. Thermogravimetric (TG) analysis was performed on a NETZSCH STA 449F3 unit at a heating rate of 10 K min^−1^ under a nitrogen atmosphere. Solid-state optical diffuse reflectance spectra were recorded on a Shimadzu 2600 UV/vis spectrometer at room temperature (RT) in the range of 800–200 nm. A BaSO_4_ plate is utilized as a standard that possesses 100% reflectance. The absorption data were obtained by converting the diffuse reflection spectrum through the Kubelka–Munk function [37]. Photoluminescence excitation (PLE) spectra, photoluminescence (PL) spectra, time-resolved PL spectra, and PLQYs were performed on an Edinburgh FLS1000 NV/V/NIR fluorescence spectrometer.

**Hirshfeld surface analyses.** The intermolecular interactions of **1** and **2** have been analyzed through Hirshfeld surface analysis via Crystal Explore 17 program [38,39,40,41,42,43]. The fabricated Hirshfeld surface of the crystal molecule is created by partitioning space in the crystal into regions. The ratio of electron density of a sum of spherical atoms in the regions for the molecule (the promolecule) is 0.5. The distance from the Hirshfeld surface to the nearest nucleus outside the surface is defined as *d_e_*. The distance from the Hirshfeld surface to the nearest internal nucleus is defined as *d_i_*. The sum of *d_e_* and *d_i_* is regarded as *d_norm_*, which is normalized by van der Waals radii (*r^vdw^*). The red highlights on the Hirshfeld surface *d_norm_* indicate that the intermolecular contacts are closer than the sum of their van der Waals radii, while the white highlights denote the interactions around the sum of *r^vdw^* and the blues represent the longer contacts. 2D fingerprint plots are employed to summarize the intermolecular interactions, which are formed by plotting the distribution of points that are derived from the Hirshfeld surface [38]. Each point in the 2D fingerprint plots corresponds to a unique (*d_e_*, *d_i_*) pair with the color referring to the contribution of the weak interactions. Red shows the greatest contribution while blue indicates the smallest contribution.

## Data Availability

All the available data are incorporated in the manuscript.

## Supplementary Materials

The following supporting information can be downloaded at: https://www.mdpi.com/article/10.3390/molecules28052380/s1. Figure S1: *ORTEP* drawing (50% ellipsoid probability) of the asymmetric unit of 1 at 100 K; Figure S2: *ORTEP* drawing (50% ellipsoid probability) of the asymmetric unit of 2 at 100 K; Figure S3: A diagram showing the hydrogen bonds (yellow dotted lines) among anionic units for 1 at 100 K; Figure S4: A diagram showing the π···π interactions (orange dotted lines) among anionic units for 1 at 100 K; Figure S5: A diagram showing the hydrogen bonds (yellow dotted lines) between the anionic and cationic units for 1 at 100 K; Figure S6: A diagram showing the π···π interactions (orange dotted lines) between the anionic and cationic units for 1 at 100 K; Figure S7: A diagram showing the anion···π interactions (green dotted lines) between the anionic and cationic units for 1 at 100 K; Figure S8: A diagram showing the hydrogen bonds (yellow dotted lines) and C-H···π interactions (red dotted lines) among anionic units for 2 at 100 K; Figure S9: A diagram showing the hydrogen bonds (yellow dotted lines) between the anionic and cationic units for 2 at 100 K; Figure S10: A diagram showing the C-H···π interactions (red dotted lines) between the anionic and cationic units for 2 at 100 K; Figure S11: The simulated and experimental PXRD patterns of 1; Figure S12: The simulated and experimental PXRD patterns of 2; Figure S13: The thermogravimetric curve for 1; Figure S14: The thermogravimetric curve for 2; Figure S15: The solid-state optical absorption spectrum of 1; Figure S16: The solid-state optical absorption spectrum of 2; Figure S17: The photographs of 1 under natural (left) and UV light (right); Figure S18: The photographs of 2 under natural (left) and UV light (right); Figure S19: The photoluminescent spectra for calculating PLQY of 1; Figure S20: The photoluminescent spectra for calculating PLQY of 2; Table S1: Crystallographic data and refinement details for 1, and 2; Table S2: Selected bond lengths (Å) and bond angles (°) for 1; Table S3: Selected bond lengths (Å) and bond angles (°) for 2; Table S4: Hydrogen bonding data for 1 at 100 K; Table S5: Hydrogen bonding data for 2 at 100 K; Table S6: The π···π interaction data for 1 at 100 K; Table S7: The anion···π interaction data for 1 at 100 K; Table S8: The C-H···π interaction data for 2 at 100 K.
