# Supplementary material for: Luminescence Enhancement and Temperature Sensing Properties of Hybrid Bismuth Halides Achieved via Tuning Organic Cations
Source: Molecules. 2023 Mar 4;28(5):2380. doi: 10.3390/molecules28052380 (PMC10005380; doi:10.3390/molecules28052380)
Supplement: Supplementary file 1 [file molecules-28-02380-s001.zip › molecules-2229355-supplementary.pdf]

Supporting Information

Luminescence Enhancement and Temperature Sensing Properties of Hybrid Bismuth Halides Achieved via Tuning Organic Cations

Ting-Hui Zhuang [1,2], Yi-Min Lin [1,2], Hao-Wei Lin [2,3], Yan-Ling Guo [1,2], Zi-Wei Li [1,2], Ke-Zhao Du [1,*], Ze-Ping Wang [2,*] and Xiao-Ying Huang [2,*]

1 Fujian Provincial Key Laboratory of Advanced Materials Oriented Chemical Engineering, College of Chemistry and Materials Science, Fujian Normal University, Fuzhou 350007, China
2 State Key Laboratory of Structural Chemistry, Fujian Institute of Research on the Structure of Matter, Chinese Academy of Sciences, Fuzhou 350002, China
3 College of Chemistry, Fuzhou University, Fuzhou 350116, China
* Correspondence: dkz007002@163.com (K.-Z.D.); wzping@fjirsm.ac.cn (Z.-P.W.); xyhuang@fjirsm.ac.cn (X.-Y.H.); Tel.: +0591-63173145 (X.-Y.H.)

Table S1. Crystallographic data and refinement details for **1**, and **2**.

Compound	**1**	**2**
Empirical formula	$C_{21}H_{22}BiCl_4N_3$	$C_{22}H_{30.50}BiCl_4N_3O_{0.25}$
Formula Mass	667.19	691.77
Crystal system	monoclinic	monoclinic
Space group	$P2_1/c$	$P2_1$
a/Å	10.1667(7)	11.3962(9)
b/Å	15.5703(7)	18.0531(15)
c/Å	15.1177(8)	12.2968(8)
α/°	90	90
β/°	105.882(7)	91.253(7)
γ/°	90	90
V/Å^3	2301.8(2)	2529.3(3)
Z	4	4
T/K	100(2)	100(2)
λ/Å	0.71073	0.71073
F(000)	1280	1346
ρ_{calcd}/g cm^{-3}	1.925	1.817
μ/mm^{-1}	8.137	7.409
Measured refls.	26752	25656
Independent refls.	5751	8866
No. of parameters	356	555
R_{int}	0.0845	0.0763
R_1 ($I > 2\sigma(I)$) [a]	0.0361	0.0416
$wR(F^2)$ ($I > 2\sigma(I)$) [b]	0.0684	0.0690
GOF	1.061	1.014

[a] $R_1 = \sum\|F_o| - |F_c\|/\sum|F_o|$, [b] $wR_2 = [\sum w(F_o^2 - F_c^2)^2/\sum w(F_o^2)^2]^{1/2}$.

Table S2. Selected bond lengths (Å) and bond angles (°) for **1**.

Selected bond lengths (Å)			
Bi(1)-N(1)	2.501(4)	Bi(1)-Cl(4)	2.7148(15)
Bi(1)-N(2)	2.528(3)	N(1)-C(1)	1.332(5)
Bi(1)-Cl(3)	2.6488(12)	N(1)-C(12)	1.360(5)
Bi(1)-Cl(2)	2.6536(12)	N(2)-C(10)	1.329(5)
Bi(1)-Cl(1)	2.6725(14)	N(2)-C(11)	1.357(5)
**Selected bond angles (°)**			
N(1)-Bi(1)-N(2)	66.05(11)	N(1)-Bi(1)-Cl(4)	85.54(9)
N(1)-Bi(1)-Cl(3)	160.23(8)	N(2)-Bi(1)-Cl(4)	88.70(9)
N(2)-Bi(1)-Cl(3)	94.19(8)	Cl(3)-Bi(1)-Cl(4)	93.84(4)
N(1)-Bi(1)-Cl(2)	94.45(8)	Cl(2)-Bi(1)-Cl(4)	94.87(4)
N(2)-Bi(1)-Cl(2)	159.90(8)	Cl(1)-Bi(1)-Cl(4)	168.54(4)
Cl(3)-Bi(1)-Cl(2)	105.28(4)	C(1)-N(1)-Bi(1)	122.4(3)
N(1)-Bi(1)-Cl(1)	83.54(9)	C(12)-N(1)-Bi(1)	118.7(3)

N(2)-Bi(1)-Cl(1)	83.71(9)	C(10)-N(2)-Bi(1)	122.7(3)
Cl(3)-Bi(1)-Cl(1)	95.26(5)	C(11)-N(2)-Bi(1)	117.9(3)
Cl(2)-Bi(1)-Cl(1)	89.44(4)		

Table S3. Selected bond lengths (Å) and bond angles (°) for **2**.

Selected bond lengths (Å)			
Bi(1)-N(2)	2.431(12)	Bi(2)-N(3)	2.469(12)
Bi(1)-N(1)	2.467(12)	Bi(2)-N(4)	2.498(12)
Bi(1)-Cl(4)	2.609(4)	Bi(2)-Cl(8)	2.649(3)
Bi(1)-Cl(3)	2.650(4)	Bi(2)-Cl(7)	2.653(4)
Bi(1)-Cl(1)	2.716(3)	Bi(2)-Cl(6)	2.686(4)
Bi(1)-Cl(2)	2.746(4)	Bi(2)-Cl(5)	2.698(3)
N(1)-C(1)	1.316(17)	N(3)-C(13)	1.297(16)
N(1)-C(12)	1.346(17)	N(3)-C(24)	1.355(17)
N(2)-C(10)	1.325(17)	N(4)-C(22)	1.286(16)
N(2)-C(11)	1.356(17)	N(4)-C(23)	1.349(18)
Selected bond angles (°)			
N(2)-Bi(1)-Cl(1)	81.6(3)	N(3)-Bi(2)-N(4)	67.2(4)
N(1)-Bi(1)-Cl(1)	78.0(3)	N(3)-Bi(2)-Cl(8)	86.6(3)
Cl(4)-Bi(1)-Cl(1)	159.14(16)	N(4)-Bi(2)-Cl(8)	83.6(3)
Cl(3)-Bi(1)-Cl(1)	96.31(11)	N(3)-Bi(2)-Cl(7)	90.2(3)
N(2)-Bi(1)-Cl(2)	161.2(3)	N(4)-Bi(2)-Cl(7)	157.2(3)
N(2)-Bi(1)-N(1)	67.8(4)	Cl(8)-Bi(2)-Cl(7)	99.18(11)
N(2)-Bi(1)-Cl(4)	82.4(3)	N(3)-Bi(2)-Cl(6)	160.3(3)
N(1)-Bi(1)-Cl(4)	83.7(3)	N(4)-Bi(2)-Cl(6)	94.7(3)
N(2)-Bi(1)-Cl(3)	89.5(3)	Cl(8)-Bi(2)-Cl(6)	99.71(12)
N(1)-Bi(1)-Cl(3)	157.1(3)	Cl(7)-Bi(2)-Cl(6)	107.02(14)
Cl(4)-Bi(1)-Cl(3)	96.79(12)	N(3)-Bi(2)-Cl(5)	77.7(3)
N(1)-Bi(1)-Cl(2)	93.4(3)	N(4)-Bi(2)-Cl(5)	80.6(3)
Cl(4)-Bi(1)-Cl(2)	96.26(12)	Cl(8)-Bi(2)-Cl(5)	161.04(16)
Cl(3)-Bi(1)-Cl(2)	109.23(13)	Cl(7)-Bi(2)-Cl(5)	91.52(11)
Cl(1)-Bi(1)-Cl(2)	94.66(12)	Cl(6)-Bi(2)-Cl(5)	91.97(13)
C(1)-N(1)-Bi(1)	123.3(10)	C(13)-N(3)-Bi(2)	124.2(10)
C(12)-N(1)-Bi(1)	116.4(8)	C(24)-N(3)-Bi(2)	117.4(9)
C(10)-N(2)-Bi(1)	123.4(10)	C(22)-N(4)-Bi(2)	123.2(10)
C(11)-N(2)-Bi(1)	117.7(8)	C(23)-N(4)-Bi(2)	116.3(8)

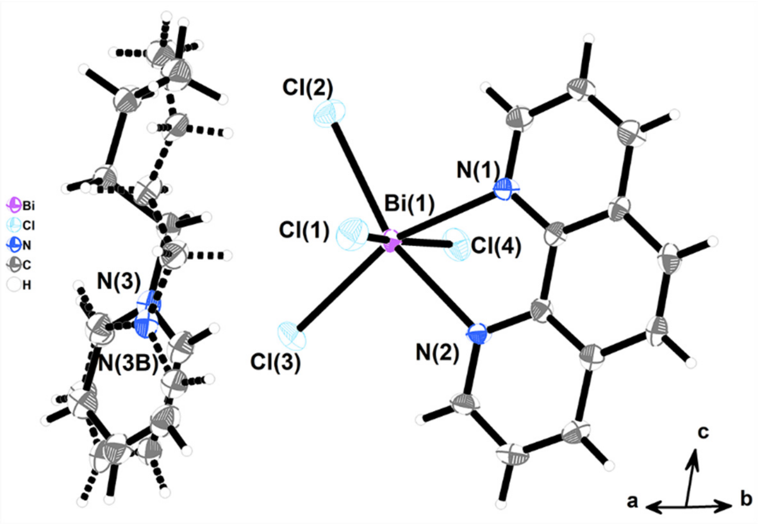

Figure S1. *ORTEP* drawing (50% ellipsoid probability) of the asymmetric unit of **1** at 100 K.

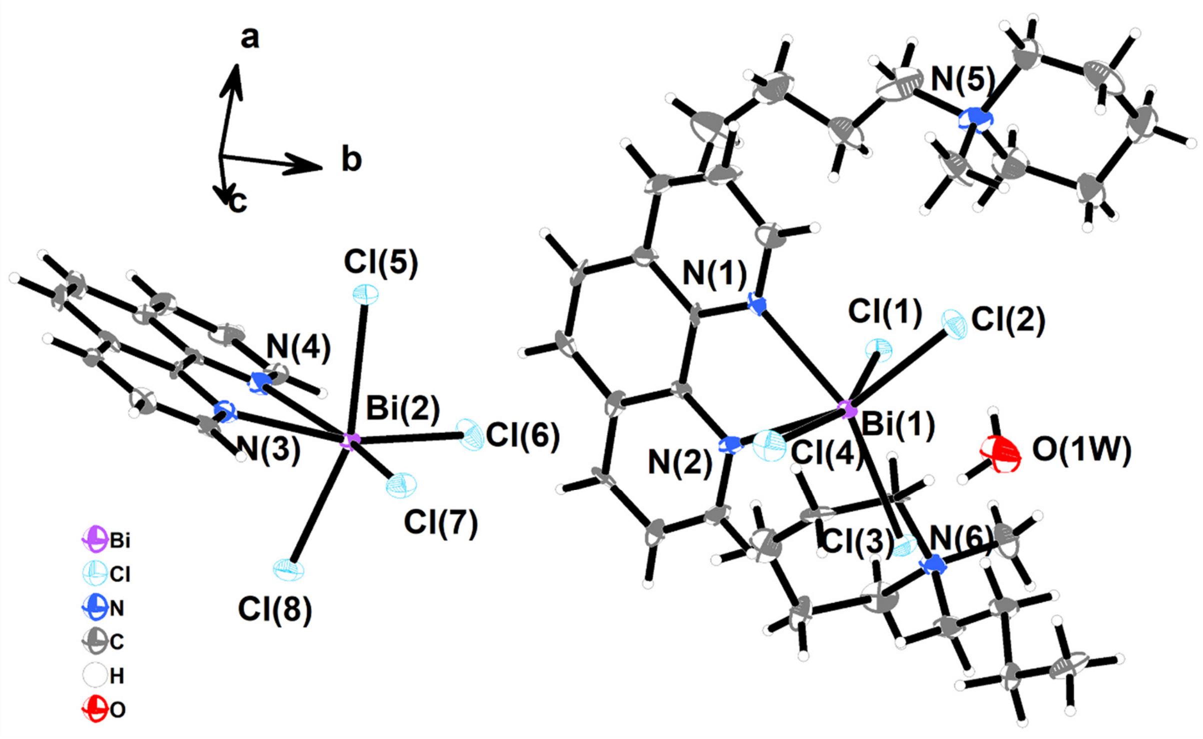

Figure S2. *ORTEP* drawing (50% ellipsoid probability) of the asymmetric unit of **2** at 100 K.

Table S4. Hydrogen bonding data for **1** at 100 K.

D–H···A	D–H (Å)	H···A (Å)	D···A (Å)	<(DHA) (°)
C(1)-H(1A)···Cl(3)#1	0.93	2.88	3.555(4)	130.2
C(10)-H(10A)···Cl(2)#2	0.93	2.99	3.466(4)	113.4
C(14^a)-H(14A^a)···Cl(2)#2	0.93	2.92	3.553(11)	126.6
C(16^a)-H(16A^a)···Cl(1)#3	0.93	2.68	3.504(11)	148.5
C(17^a)-H(17A^a)···Cl(2)#4	0.93	2.64	3.493(18)	153.1
C(18^a)-H(18B^a)···Cl(1)	0.97	2.87	3.768(9)	154.6
C(14B^b)-H(14B^b)···Cl(1)#2	0.93	2.75	3.593(13)	151

C(16B^b)-H(16B^b)···Cl(2)#4	0.93	2.99	3.572(14)	121.8
C(17B^b)-H(17B^b)···Cl(2)#4	0.93	2.86	3.50(3)	126.9
C(18B^b)-H(18C^b)···Cl(1)	0.97	2.53	3.488(17)	168.7
C(18B^b)-H(18D^b)···Cl(4)#5	0.97	2.52	3.485(18)	178.9

Symmetry transformations used to generate equivalent atoms: #1 x,-y+1/2,z+1/2; #2 x,-y+1/2,z-1/2; #3 -x+1,y-1/2,-z+1/2; #4 -x+1,-y,-z+1; #5 x+1,y,z.

Table S5. Hydrogen bonding data for **2** at 100 K.

D–H···A	D–H (Å)	H···A (Å)	D···A (Å)	<(DHA) (°)
C(1)-H(1)···Cl(5)#1	0.95	2.82	3.518(15)	130.6
C(2)-H(2)···Cl(5)#1	0.95	2.98	3.586(18)	122.9
C(10)-H(10)···Cl(3)	0.95	2.81	3.502(16)	130.5
C(10)-H(10)···Cl(8)#2	0.95	2.94	3.623(14)	130
C(13)-H(13)···Cl(4)#3	0.95	2.76	3.500(13)	135.5
C(13)-H(13)···Cl(7)	0.95	2.9	3.580(16)	129.9
C(22)-H(22)···Cl(1)#4	0.95	2.85	3.574(14)	134
C(25)-H(25A)···Cl(3)#5	0.99	2.89	3.806(17)	154.2
C(25)-H(25B)···Cl(7)#1	0.99	2.71	3.673(16)	164.3
C(29)-H(29B)···Cl(6)#6	0.99	2.92	3.544(16)	122.3
C(30)-H(30B)···Cl(1)	0.98	2.82	3.762(16)	161.1
C(30)-H(30C)···Cl(5)#1	0.98	2.9	3.830(17)	157.7
C(31)-H(31B)···Cl(3)#5	0.99	2.9	3.798(19)	152
C(35)-H(35A)···Cl(5)#6	0.99	2.87	3.784(13)	153.6
C(38)-H(38A)···Cl(1)	0.99	2.99	3.665(14)	126.4
C(38)-H(38B)···Cl(8)#2	0.99	2.99	3.865(15)	148.1
C(40)-H(40B)···Cl(5)#6	0.98	2.74	3.679(18)	159.6
C(41)-H(41A)···Cl(8)#2	0.99	2.93	3.910(16)	168.8
C(41)-H(41B)···Cl(7)#2	0.99	2.81	3.499(15)	127.5
C(42)-H(42A)···Cl(3)	0.99	2.9	3.654(14)	133.3
O(1W)-H(1B)···Cl(2)	0.82	2.51	3.26(2)	152.8
O(1W)-H(1A)···Cl(7)#7	0.82	2.7	3.33(2)	134.8

Symmetry transformations used to generate equivalent atoms: #1 -x+1,y+1/2,-z; #2 -x,y+1/2,-z+1; #3 -x,y-1/2,-z; #4 -x+1,y-1/2,-z+1; #5 x+1,y,z; #6 -x+1,y+1/2,-z+1; #7 -x,y+1/2,-z.

Table S6. The π···π interaction data for **1** at 100 K.

Cg((I)···Cg((J)	ARU(J)	Cg···Cg(Å)	α(°)	β(°)	γ(°)
Cg(2)→Cg(2)	[3566.01]	3.669(3)	0.0(2)	22.4	22.4
Cg(2)→Cg(4)	[3566.01]	3.798(3)	1.2(2)	27.6	26.7
Cg(3)→Cg(5)	[2655.02]	3.750(5)	9.1(5)	21.1	29.8
Cg(3)→Cg(6)	[2655.03]	3.603(14)	5	19.6	23.8
Cg(4)→Cg(2)	[3566.01]	3.799(3)	1.2(2)	26.7	27.6
Cg(4)→Cg(6)	[2655.03]	3.963(14)	4	30.9	29.8

Cg(5)→Cg(3)	[2645.01]	3.750(5)	9.1(5)	29.8	21.1
Cg(6)→Cg(3)	[2645.01]	3.604(14)	5	23.8	19.6
Cg(6)→Cg(4)	[2645.01]	3.963(14)	4	29.8	30.9

[3566] = -X,1-Y,1-Z; [2655] = 1-X,1/2+Y,1/2-Z; [2655] = 1-X,1/2+Y,1/2-Z; [2645] = 1-X,-1/2+Y,1/2-Z;
Cg(2): N(1)→C(1)→C(2)→C(3)→C(4)→C(5)
Cg(3): N(2)→C(11)→C(7)→C(8)→C(9)→C(10)
Cg(4): C(4)→C(5)→C(6)→C(7)→C(11)→C(12)
Cg(5): N(3A)→C(13A)→C(14A)→C(15A)→C(16A)→C(17A)
Cg(6): N(3B)→C(13B)→C(14B)→C(15B)→C(16B)→C(17B)

Table S7. The anion···π interaction data for **1** at 100 K.

Y-X(I)→Cg(J)	ARU(J)	X···Cg(Å)	γ(°)
Bi(1)-Cl(3)→Cg(5)	[1555.02]	3.548(5)	23.71
Bi(1)-Cl(3)→Cg(6)	[1555.03]	3.727(15)	19.76

[1555] = X,Y,Z

Table S8. The C-H···π interaction data for **2** at 100 K.

C-H(I)→Cg(J)	ARU(J)	X···Cg(Å)	γ(°)
C(2)-H(2)→Cg6	[2655.02]	2.93	14.51
C(21)-H(21)→Cg4	[2646.01]	2.88	16.45
C(26)-H(26A)→Cg4	[2655.01]	2.64	10.91
C(44)-H(44A)→Cg6	[1565.02]	2.9	13.21

[2655] = 1-X,1/2+Y,-Z; [2646] = 1-X,-1/2+Y,1-Z; [1565] = X,1+Y,Z;
Cg(4): C(4)→C(5)→C(6)→C(7)→C(11)→C(12)
Cg(6): N(3)→C(13)→C(14)→C(15)→C(16)→C(24)

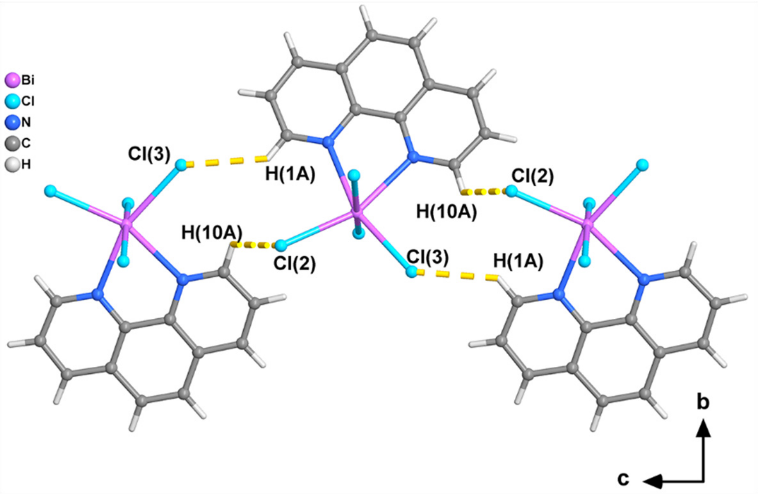

Figure S3. A diagram showing the hydrogen bonds (yellow dotted lines) among anionic units for **1** at 100 K.

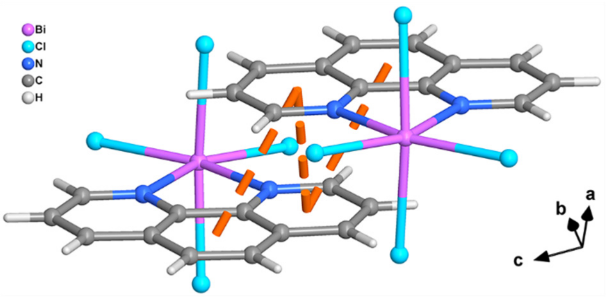

Figure S4. A diagram showing the π···π interactions (orange dotted lines) among anionic units for **1** at 100 K.

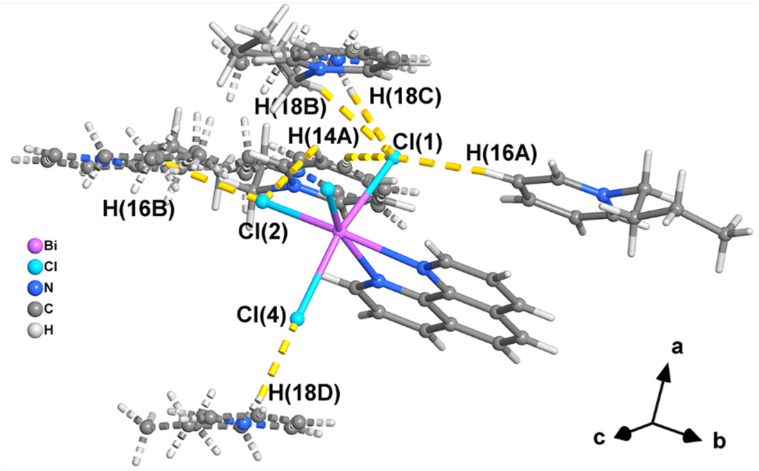

Figure S5. A diagram showing the hydrogen bonds (yellow dotted lines) between the anionic and cationic units for **1** at100 K.

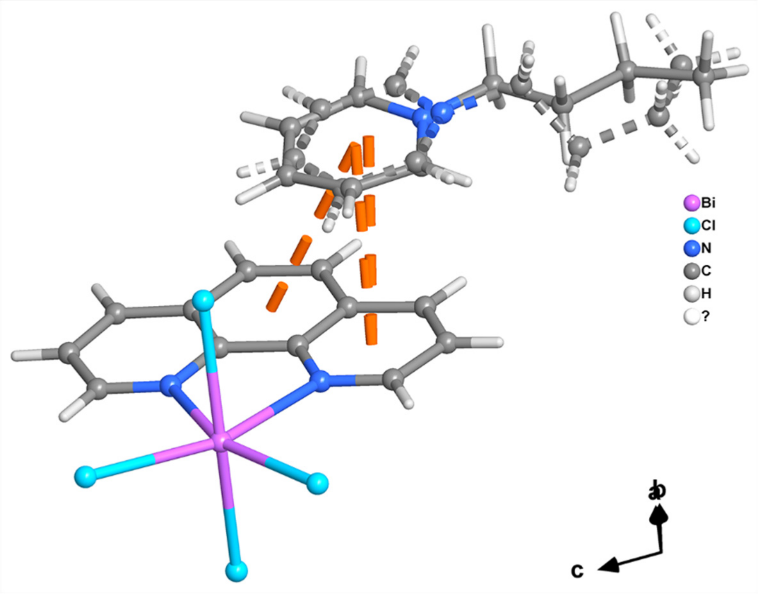

Figure S6. A diagram showing the π···π interactions (orange dotted lines) between the anionic and cationic units for **1** at 100 K.

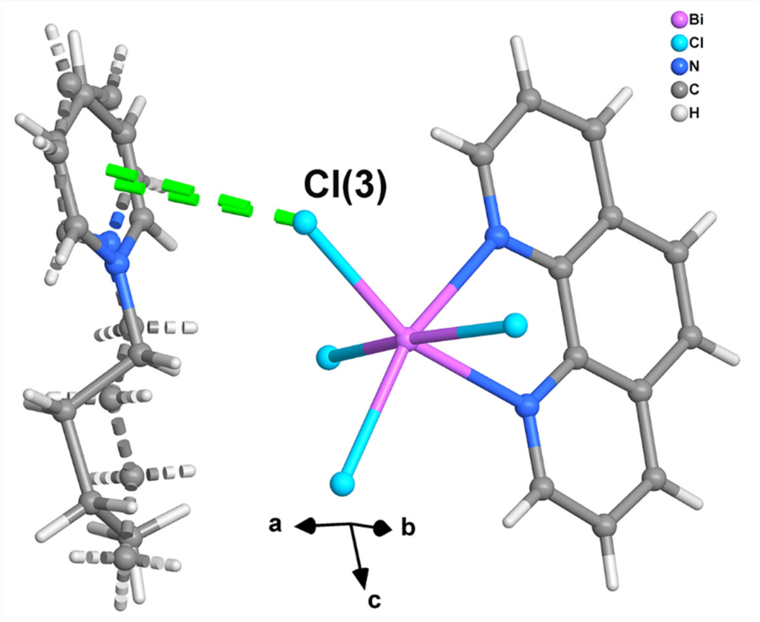

Figure S7. A diagram showing the anion···π interactions (green dotted lines) between the anionic and cationic units for **1** at 100 K.

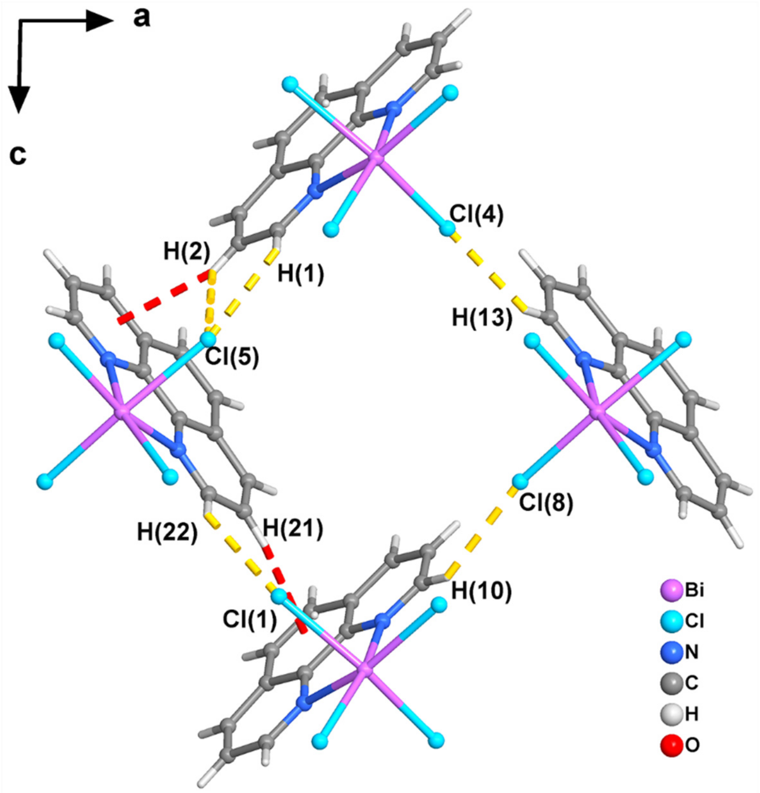

Figure S8. A diagram showing the hydrogen bonds (yellow dotted lines) and C-H···π interactions (red dotted lines) among anionic units for **2** at 100 K.

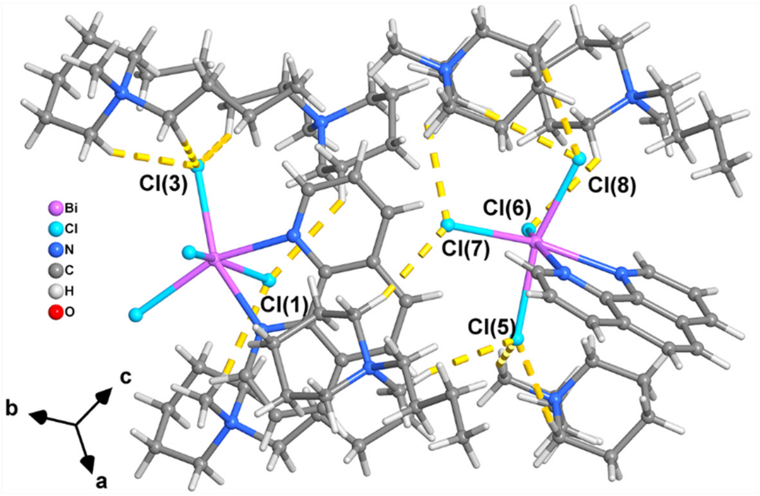

Figure S9. A diagram showing the hydrogen bonds (yellow dotted lines) between the anionic and cationic units for **2** at 100 K.

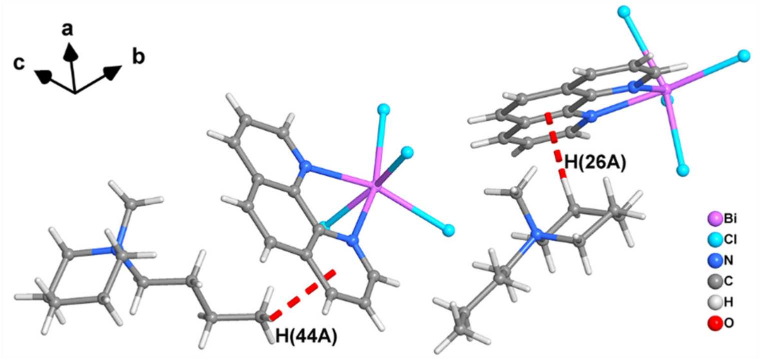

Figure S10. A diagram showing the C-H···π interactions (red dotted lines) between the anionic and cationic units for **2** at 100 K.

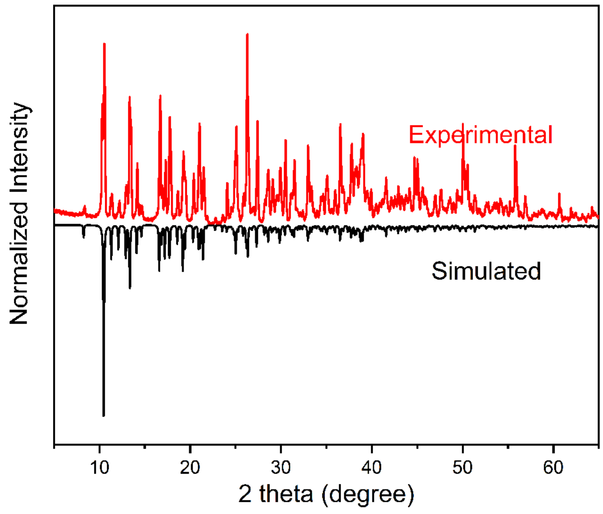

Figure S11. The simulated and experimental PXRD patterns of **1**.

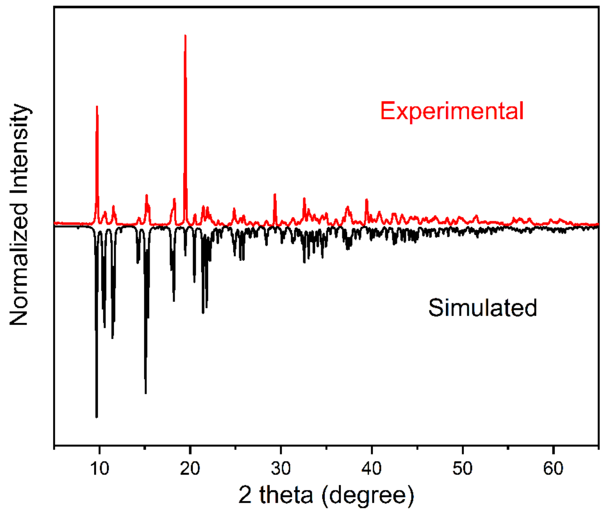

Figure S12. The simulated and experimental PXRD patterns of **2**.

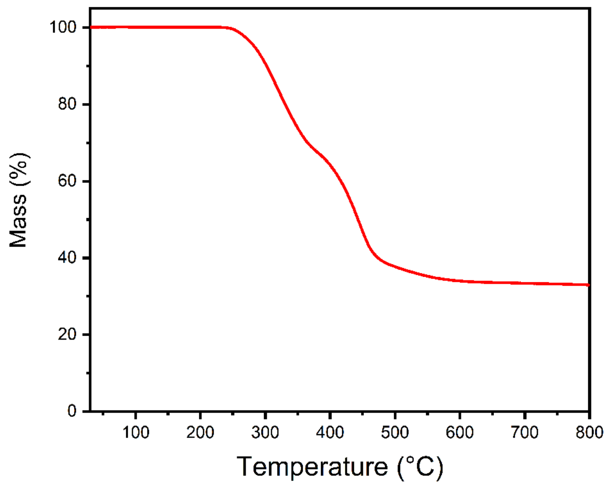

Figure S13. The thermogravimetric curve for **1**.

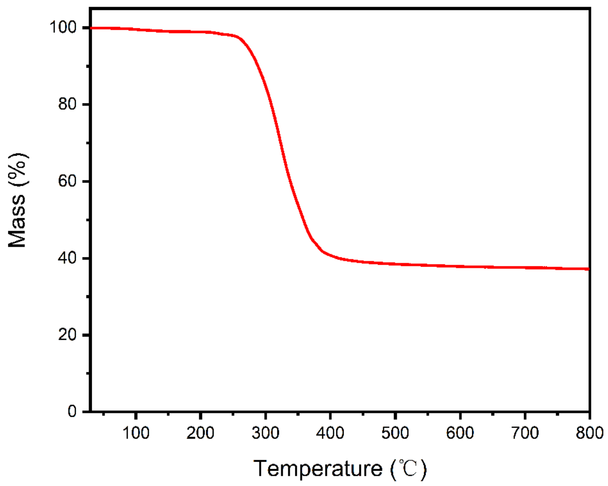

Figure S14. The thermogravimetric curve for **2**.

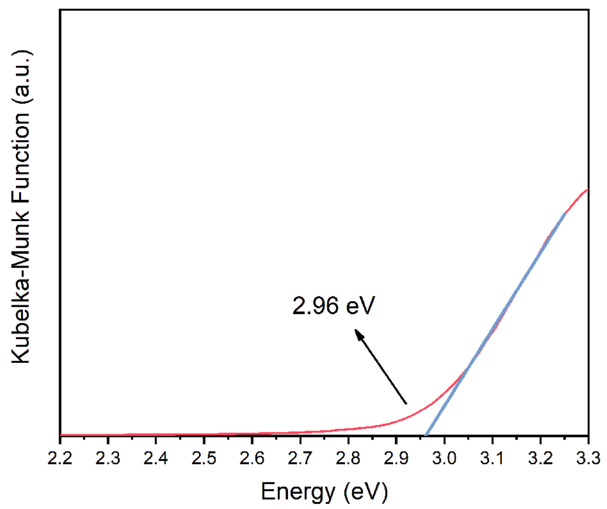

Figure S15. The solid-state optical absorption spectrum of **1**.

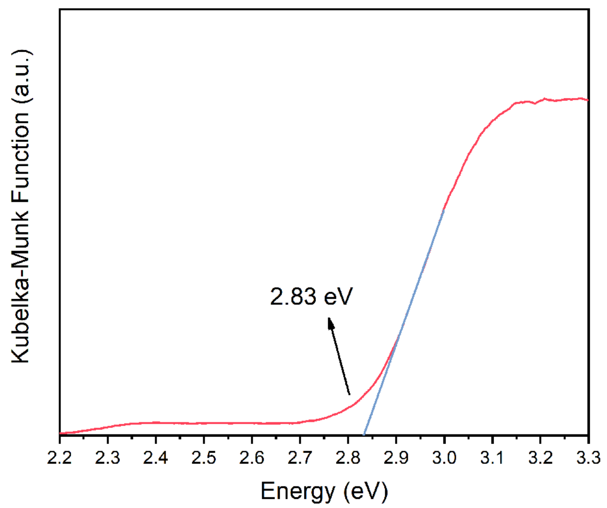

Figure S16. The solid-state optical absorption spectrum of **2**.

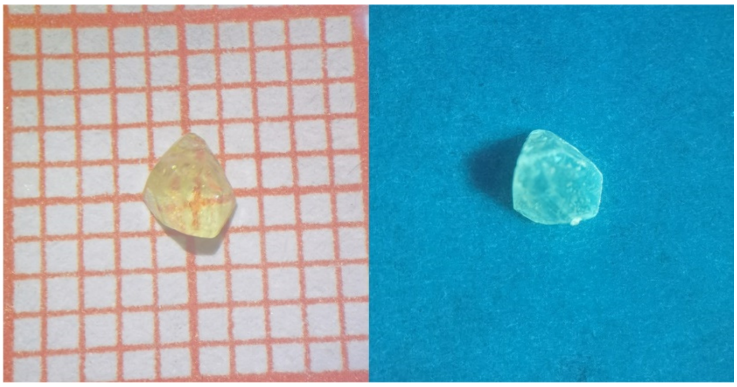

Figure S17. The photographs of **1** under natural (left) and UV light (right).

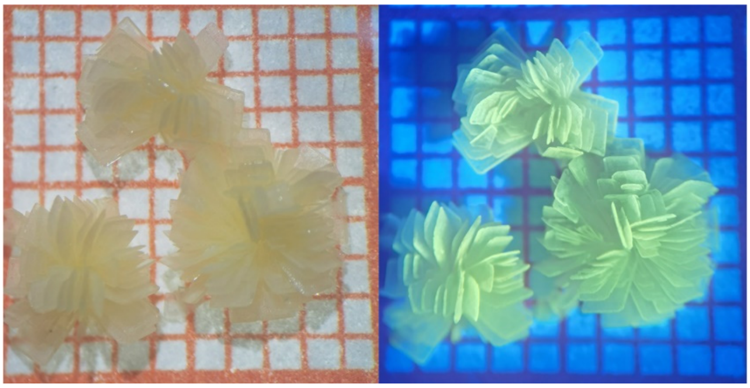

Figure S18. The photographs of **2** under natural (left) and UV light (right).

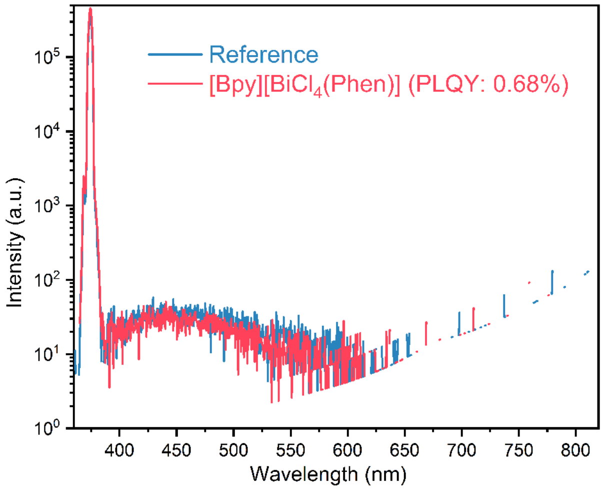

Figure S19. The photoluminescent spectra for calculating PLQY of **1**.

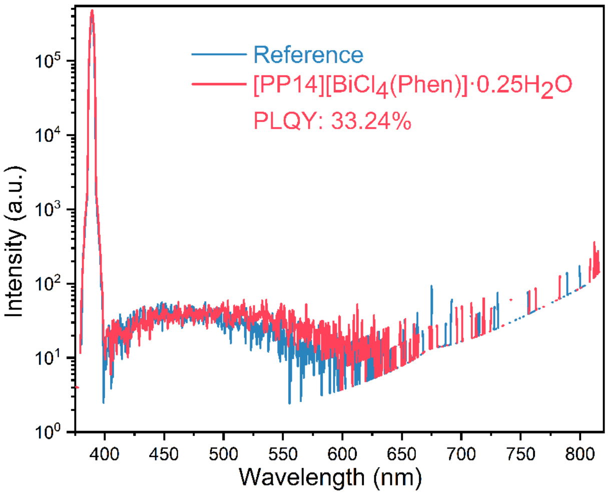

Figure S20. The photoluminescent spectra for calculating PLQY of **2**.
